# The mediating role of self-perceived health and fitness in the relationship between physical activity and quality of life among individuals with physical disabilities: the moderating effect of self-esteem

**DOI:** 10.3389/fpsyg.2026.1784783

**Published:** 2026-08-27

**Authors:** Majed M. Alhumaid

**Affiliations:** Department of Physical Education, College of Education, King Faisal University, Al-Ahsa, Saudi Arabia

**Keywords:** physical activity, physical disability, psychological well-being, quality of life, self-esteem, self-perceptions

## Abstract

**Background:**

This study sought to investigate the psychological mechanisms that connect physical activity (PA) to quality of life (QoL) in individuals with physical disabilities (IWPDs). Three hypotheses were tested: (1) self-perceived health and self-perceived fitness mediate distinct indirect associations involving PA, self-perceptions, and QoL; (2) self-esteem moderates the indirect association between PA and QoL through self-perceived fitness; and (3) an integrated model, including PA level, self-perceptions, and self-esteem, explains a significant variance in QoL.

**Methods:**

A cross-sectional study was performed involving 230 individuals with physical disabilities. Measures comprised PA level (Arabic version of the Physical Activity Scale for Individuals with Physical Disabilities), self-perceived fitness and health, self-esteem (Rosenberg Self-Esteem Scale), QoL (World Health Organization Quality of Life–Disabilities module), and demographic covariates. Mediation and moderated mediation analyses were conducted using the mediation package in R, employing 1,000 bias-corrected bootstrap resamples. Hypothesis 3 was evaluated through hierarchical regression and structural equation modeling using the lavaan package. All tests were checked for validity and reliability among all participants before analyses.

**Results:**

Self-perceived health significantly mediated the relationship between self-perceived fitness and QoL (average causal mediation effect = 0.123, 95% CI [0.071, 0.179], *p* < 0.001), representing 42.3% of the total effect. Self-perceived fitness served as a partial mediator in the relationship between PA level and QoL (average causal mediation effect = 0.004, 95% CI [0.002, 0.007], *p* < 0.001). Additionally, this mediation effect was moderated by self-esteem, with the influence increasing from low to high self-esteem groups. In the integrated model, self-perceived health (*β* = 0.431) and fitness (*β* = 0.203) emerged as the only significant predictors: PA level and self-esteem showed small, statistically non-significant direct associations with QoL.

**Conclusion:**

The findings endorse the perceptual pathways model, indicating that cognitive appraisals, specifically self-perceived fitness and self-perceived health, are involved in distinct indirect associations between PA and QoL, with self-esteem serving as a moderating factor. Interventions should focus on improving self-perceptions while also encouraging PA, as subjective evaluations showed stronger statistical associations with QoL than the reported PA level in IWPDs.

## Introduction

### Context and importance

Quality of life (QoL) is a multidimensional construct central to health and rehabilitation, encompassing physical, psychological, and social domains that reflect how individuals perceive their position in life relative to personal goals, values, and sociocultural expectations (World Health Organization, 2012). For individuals with physical disabilities (IWPDs), achieving optimal QoL is a complex challenge. Research consistently shows that compared with their peers without disabilities, IWPDs report lower QoL across physical health, psychological well-being, social participation, and environmental access (Ekediegwu et al., 2024; Ibsen et al., 2023). These disparities arise from factors such as functional limitations, environmental barriers, limited employment and recreational opportunities, and social stigma (Alhumaid and Said, 2025). Additionally, psychosocial factors, including reduced self-efficacy, social isolation, and experiences of discrimination, further diminish life satisfaction (Yang et al., 2022).

In Saudi Arabia, these challenges intersect with unique cultural and structural factors. Historically, IWPDs have faced limited public accessibility, underdeveloped inclusive infrastructure, and low representation in community and employment settings (Alhazmi et al., 2025). Although initiatives under Vision 2030 promote social inclusion, equitable healthcare access, and community participation, disparities in QoL remain (Said and Alhumaid, 2024). Many Saudi IWPDs experience restricted opportunities for physical activity (PA), limited mobility, and reduced social engagement, undermining both physical and psychological well-being (Martin Ginis, 2025). Cultural norms emphasizing familial protection may also limit autonomy and active lifestyle participation, further affecting QoL (White et al., 2024).

Identifying modifiable behavioral and perceptual factors that enhance QoL among Saudi IWPDs is therefore a critical public health and rehabilitation priority. PA is a particularly promising avenue, offering benefits that extend beyond functional improvement to include positive self-perceptions and psychosocial resilience.

### Physical activity and mechanisms of well-being

Physical activity is a central determinant of QoL across populations with and without disabilities (Keramat et al., 2022; White et al., 2024). Among IWPDs, regular PA improves physical health, psychological well-being, social integration, and life satisfaction (Chen et al., 2025; Joensuu et al., 2024). The mechanisms underlying these benefits are multifaceted, encompassing physiological, psychological, and social dimensions.

Physiologically, PA enhances cardiovascular efficiency, muscular strength, flexibility, and coordination, promoting functional independence and reducing fatigue (Ekediegwu et al., 2024). These improvements mitigate secondary health complications, such as obesity, joint pain, and metabolic disorders, which commonly co-occur with disability and negatively affect QoL (Martin Ginis, 2025). Additionally, improved functional ability strengthens perceived control over daily life, fostering autonomy and competence.

Psychologically, PA activates neurobiological processes that reduce stress and depressive symptoms via endorphin, dopamine, and serotonin release while enhancing cognitive function and mood regulation (Rodriguez-Ayllon et al., 2023). These effects support emotional stability, self-efficacy, and self-acceptance, critical elements of subjective well-being (Chen et al., 2025). PA also offers IWPDs opportunities for mastery and positive body awareness, countering dependence and social stigma (Yang et al., 2022).

Socially, structured or group-based PA fosters connectedness, recognition, and belonging, addressing key psychosocial barriers such as isolation and marginalization (White et al., 2024). Despite these benefits, most research focuses on direct PA–QoL associations, leaving the psychological and perceptual pathways underexplored. Therefore, understanding how self-perceptions of health and fitness mediate the effects of PA on QoL is essential, particularly in culturally specific contexts such as Saudi Arabia.

### Mediating role of self-perceived health and fitness

The Wilson and Cleary (1995) model links behavioral and clinical factors to perceived health and overall QoL, highlighting the importance of self-perceptions. Self-perceived (SP) health and fitness are key psychological appraisals that mediate the impact of PA on QoL (Pasanen et al., 2014; Rodriguez-Ayllon et al., 2023). PA enhances QoL through interconnected physiological, cognitive, and emotional pathways. Physically, it strengthens muscles; improves endurance and cardiovascular efficiency; boosts flexibility, coordination, and functional independence, thereby facilitating everyday tasks (Schwartz et al., 2025). Cognitively, these changes are appraised positively, fostering better self-perceptions of health and fitness, a stronger sense of control, and increased competence. Emotionally, this positive appraisal cultivates greater well-being, higher life satisfaction, more positive affect, and enhanced resilience to stress (Yerrakalva et al., 2023). Together, these mechanisms explain how regular PA translates into meaningful, holistic improvements in overall QoL.

*H1:* Self-perceived health and self-perceived fitness will function as mediators in distinct indirect associations, including (a) the association between PA level and overall QoL through SP health, (b) the association between PA level and overall QoL through SP fitness, and (c) the association between SP fitness and overall QoL through SP health.

### Moderating role of self-esteem

The strength of these pathways likely varies according to individual differences in self-esteem, which is defined as a global evaluation of personal worth (Rosenberg, 1965). Among IWPDs, self-esteem buffers against stigma and negative self-perceptions (Alhumaid et al., 2024a). According to Fredrickson’s (2001) broaden-and-build theory, individuals with higher self-esteem are more capable of integrating positive experiences, such as functional gains from PA, into lasting psychological benefits. Empirical evidence supports self-esteem as a moderator of health behaviors and QoL outcomes among individuals with disabilities (Martin Ginis, 2025; Yang et al., 2022).

*H2:* Self-esteem will moderate the indirect associations between PA level and overall QoL through self-perceived fitness, such that the strength of this indirect association varies across levels of self-esteem.

Although PA and QoL have been extensively studied, no prior research has tested a moderated mediation model incorporating health perceptions and self-esteem within the Saudi context. Cultural norms and societal attitudes toward disability influence self-concept, health behaviors, and psychosocial outcomes (Alhazmi et al., 2025). This study examines an integrated model in which PA level is the primary predictor, SP fitness and SP health are examined as mediating variables, self-esteem is examined as a moderator of the SP-fitness pathway, and QoL is the outcome among Saudi IWPDs.

*H3:* An integrated multivariable model incorporating PA level, SP fitness, SP health, and self-esteem will account for significant variance in overall QoL among IWPDs.

Using a moderated mediation design, this study provides nuanced insights into how PA enhances well-being and informs interventions to improve both physical and psychological resources.

## Methodology

### Study design

This study employed a cross-sectional, questionnaire-based design to examine the mediating roles of SP fitness and SP health in the relationship between PA levels and QoL among Saudi IWPDs. In addition, self-esteem was examined as a psychosocial variable in relation to PA and QoL. Data were collected via an online survey administered between 1 March and 30 April 2025.

### Participants and recruitment

Participants were recruited from rehabilitation centers, disability organizations, and community support networks across six administrative regions of Saudi Arabia: Riyadh, the Western Region, the Northern Border, Hail, Jazan, and Tabuk. Recruitment was conducted through direct communication by phone, email, or WhatsApp. Individuals who agreed to participate were provided with an invitation link directing them to the Google Forms survey. On the survey’s landing page, participants were informed about study objectives, the voluntary nature of participation, the estimated completion time (approximately 25 min), and their right to withdraw at any point without penalty. Then, they were required to review and electronically sign the informed consent form before accessing the questionnaire. Upon submission, responses were exported and examined for accuracy and completeness. No personally identifiable information was collected.

Eligibility criteria required participants to: (1) be at least 18 years old; (2) be fluent in Arabic; (3) have a physical disability officially recognized by the Saudi Ministry of Human Resources and Social Welfare; and (4) provide informed consent. Individuals with cognitive impairments or severe mental health conditions that could compromise their ability to complete the questionnaire were excluded. During data quality screening, responses were removed if more than 20% of scale items were missing, if response patterns were invariant, or if demographic information was contradictory (e.g., inconsistencies between age and marital or educational status). Of the 250 IWPDs invited, 243 submitted the online survey. After applying the exclusion criteria, 230 valid responses were retained for statistical analysis, yielding a completion rate of 92.0%.

### Measures

#### Demographic questionnaire

The demographic questionnaire collected information on participants’ age, gender, educational level, type of physical disability, types of physical aids used, height, weight, marital status, occupation, and average family income. In addition, the questionnaire assessed SP health and SP fitness. These variables were measured using two single-item self-assessment questions. Responses were recorded on a 3-point Likert scale (SP health: *poor*, *moderate*, or *good*; SP fitness: *low*, *moderate*, or *high*), with higher categories indicating more favorable self-perceptions. Single-item measures were selected because they provide simple, low-burden indicators of perceived health and fitness that have been widely used in epidemiological and population-based research.

#### Physical activity

PA was assessed using the Arabic version of the Physical Activity Scale for Individuals with Physical Disabilities (PASIPD-AR), a culturally adapted and validated version of the original PASIPD that estimates weekly PA through MET-based scoring (Alhumaid et al., 2024b; Washburn et al., 2002). The 13-item scale evaluates PA across four domains—leisure, home, occupation/transportation, and inactivity—based on activities performed in the preceding 7 days. Each item records frequency (never/seldom = 1–2 days/week, occasionally = 3–4 days/week, often = 5–7 days/week) and duration (<1 h, 1–2 h, 2–4 h, >4 h per day). The PASIPD-AR includes four latent factors: home repairs, lawn, and garden activities (Items 9–11); household activities (Items 7, 8, 12); sports and recreational activities (Items 3–6); and occupational and transportation activities (Items 2, 13). Item 1 serves as a contextual reference and is excluded from scoring. Total scores are calculated by multiplying average daily hours by the corresponding MET values (range: 0.0–199.5 MET-h/day), with higher scores reflecting greater activity levels.

#### Quality of life

QoL was measured using the Arabic World Health Organization Quality of Life–Disabilities module (WHOQOL-DIS), adapted to the Saudi context and validated by Said and Alhumaid (2025). The instrument contains the WHOQOL-BREF core module and the Disabilities module. Consistent with the Arabic validation study, Item 21 was excluded because of cultural considerations. The WHOQOL-BREF assesses physical health (7 items), psychological health (6 items), social relationships (2 items), and environment (8 items). The Disabilities module comprises three facets: discrimination (Items 28–30), autonomy (Items 31–33), and inclusion/participation (Items 34–39). Items 1, 2, and 27 were excluded from factor analyses because of their general nature, consistent with the original validation study. All items were rated on a 5-point Likert scale, with negatively worded items reverse-scored.

#### Self-esteem

Self-esteem was assessed using the Arabic version of the Rosenberg Self-Esteem Scale (RSES; Alhumaid, 2024), a validated 10-item self-report instrument rated on a 4-point Likert scale (1 = strongly disagree to 4 = strongly agree). Five negatively worded items were reverse-coded, and total scores ranged from 10 to 40, with higher scores indicating greater self-esteem. Following Alhumaid (2024), self-esteem was categorized into three levels: low (1.00–2.00), moderate (2.01–3.00), and high (3.01–4.00). This categorization also ensured adequate subgroup sizes for the moderated mediation analyses. The Arabic RSES has demonstrated a two-factor structure (positive and negative self-feelings). Although Item 8 showed a low factor loading in the validation study, all 10 items were retained to preserve the original scale structure and scoring.

#### Psychometric properties of the scales

Validity and reliability of all instruments were assessed in the current sample (*N* = 230). Factor analyses supported the expected structures: PASIPD-AR (four-factor; Kaiser–Meyer–Olkin [KMO] = 0.836; Bartlett’s χ^2^[66] = 1039.97, *p* < 0.001), WHOQOL-BREF (four-factor; KMO = 0.832; Bartlett’s χ^2^[253] = 4544.64, *p* < 0.001), WHOQOL Disabilities module (three-factor; KMO = 0.831; Bartlett’s χ^2^[66] = 2325.61, *p* < 0.001), and RSES (2-factor; KMO = 0.731; Bartlett’s χ^2^[45] = 1230.67, *p* < 0.001). Internal consistency was acceptable to excellent across all scales in this sample: PASIPD-AR (*α* = 0.802, domains α = 0.706–0.912), WHOQOL-BREF (α = 0.902, domains α = 0.846–0.912), WHOQOL Disabilities module (α = 0.766; domains α = 0.664–0.950), and RSES (α = 0.801; component α = 0.795–0.844). These results confirm the suitability of the measures for assessing PA, QoL, and self-esteem among IWPDs.

### Ethical considerations

The study protocol received approval from the King Faisal University Research Ethics Committee (Approval No. KFU-REC-2024-OCT-ETHICS2863). All procedures complied with the ethical standards of the Declaration of Helsinki. Participation was voluntary, and respondents could withdraw at any time. Data confidentiality and anonymity were strictly maintained.

### Statistical procedures

Statistical analyses were conducted using R version 4.5.1 (R Core Team, 2025), with the lavaan package (v0.6–19) for structural equation modeling, the mediation package for mediation analyses, the psych package for descriptive and psychometric analyses, the car package for assumption testing, the performance and semTools packages for model evaluation and validation, and ggplot2 for data visualization. Given the cross-sectional design of the study, the mediation analyses were interpreted as statistical models evaluating hypothesized indirect associations rather than as evidence of temporal or causal mechanisms.

Composite scores were computed for relevant scales. Data assumptions were checked: multivariate outliers were identified via Mahalanobis distance and winsorized at the 1st and 99th percentiles; multicollinearity was evaluated with variance inflation factors (<5), and univariate normality via skewness, kurtosis, and Q–Q plots.

Prior to analyses, the psychometric properties of all instruments (PASIPD-AR, WHOQOL-BREF, WHOQOL Disabilities module, and RSES) were evaluated in the current sample. KMO and Bartlett’s tests confirmed factorability, factor analyses supported structural validity, and internal consistency (Cronbach’s alpha) was acceptable to excellent. Specifically, alpha values for the four instruments were 0.802, 0.902, 0.766, and 0.801, respectively, with subscale values ranging from 0.664 to 0.950.

Confirmatory factor analysis was used to evaluate the four-domain structure of the Arabic WHOQOL-DIS (physical health, psychological health, social relationships, and environment), with the comparative fit index ≥ 0.90, Tucker–Lewis index ≥ 0.90, root mean square error of approximation ≤ 0.08, standardized root mean square residual < 0.08, and factor loadings > 0.40. Structural equation modeling then regressed latent QoL on demographic covariates, self-perceptions, and PA level using the diagonally weighted least squares estimator.

For mediation and moderated mediation analyses, 1,000 bias-corrected bootstrap resamples were used, with age and gender included as covariates. For Hypothesis 1, three mediation models were tested: (1) PA level → SP health → QoL; (2) PA level → SP fitness → QoL; and (3) SP fitness → SP health → QoL. For Hypothesis 2, the prespecified conditional indirect association of PA level with overall QoL through SP fitness was examined at low, moderate, and high levels of self-esteem. Indirect and conditional indirect effects were considered statistically significant when their 95% bootstrap confidence intervals excluded zero.

For Hypothesis 3, hierarchical regression examined sequential predictor blocks (PA level, self-perceptions, self-esteem, and interactions), with Δ*R*2 and *F*-tests evaluating model improvement. Predictive robustness was assessed via 70/30 cross-validation (*R*2, root mean squared error, mean absolute error, and mean absolute percentage error). All tests used *α* = 0.05. Figures, including mediation diagrams and regression plots, were generated with ggplot2, and the final conceptual model was refined using AI-assisted diagramming software.

**Figure 1:**
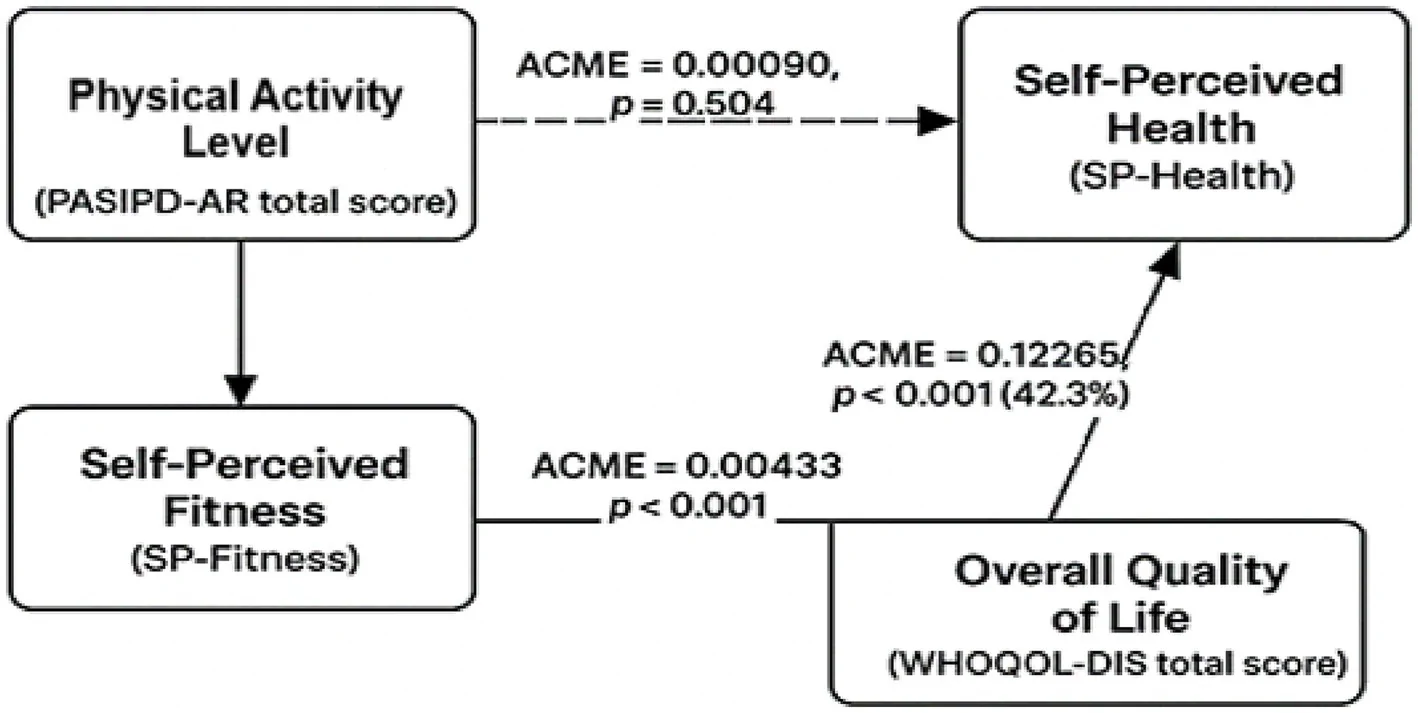
Visual summary of the three distinct component mediation pathways examined in this study: (1) PA level → SP-health → QoL; (2) PA level → SP-fitness → QoL; and (3) SP-fitness → SP-health → QoL. The full unified indirect effect from PA level through both SP-fitness and SP-health was not estimated as a single model; these pathways represent separate statistical tests. The figure synthesizes the results of these piecewise analyses for conceptual clarity.

To distinguish confirmatory from exploratory analyses, the three mediation pathways specified in Hypothesis 1 and the moderated indirect association specified in Hypothesis 2 were treated as primary analyses. Secondary analyses examined individual QoL dimensions, including domain-specific mediation (Supplementary Table S1), domain-specific moderated mediation (Figure 1), and interaction terms in hierarchical regression (Model 4). Because these analyses were exploratory, no global correction for multiple comparisons was applied. Nevertheless, the potential for inflated Type I error was acknowledged, and secondary findings were interpreted cautiously, emphasizing overall patterns of association rather than individual *p*-values.

## Results

### Hypothesis 1: mediation pathways involving self-perceived health and fitness

Hypothesis 1 proposed that self-perceived health and self-perceived fitness would function as mediating variables in the associations among PA levels and overall QoL. Three mediation pathways were examined: PA level → SP health → QoL, PA level → SP fitness → QoL, and SP fitness → SP health → QoL.

To test this hypothesis, mediation analyses were conducted using data from the full sample (*N* = 230). Three specific pathways were examined: (1) PA level → SP health → QoL (Model 1), (2) PA level → SP fitness → QoL (Model 2), and (3) SP fitness → SP health → QoL (Model 3). Analyses were performed using the mediation package in R with 1,000 bias-corrected bootstrap resamples. Additional models included age and gender as covariates. The primary analyses focused on overall QoL, while mediation effects across individual QoL domains were examined as secondary analyses.

Distinct patterns emerged across the tested pathways (Table 1). The indirect effect of the PA level on overall QoL through SP health was not statistically significant (*p* = 0.504), indicating a lack of mediation. By contrast, SP fitness significantly mediated the association between PA level and QoL, although the effect size was small (*p* < 0.001). A notable indirect association was also found between SP fitness and QoL, through SP health. This indirect effect was large and statistically significant (*p* < 0.001), accounting for 42.34% of the total effect. When age and gender were included as covariates, the indirect effect for Model 1 remained non-significant (*p* = 0.280), suggesting that demographic factors did not meaningfully alter the observed mediation patterns.

**Table 1:** Serial and parallel mediation effects of the physical activity level on overall quality of life via self-perceived fitness and self-perceived health.

Model pathway	ACME	95% CI	*p*-value	Effect size	Proportion mediated
PA level → SP-Health → Overall QoL	0.00090	[−0.00235, 0.00358]	0.504	Small	−4.98%
PA level → SP-Fitness → Overall QoL	0.00433	[0.00237, 0.00684]	<0.001	Small	23.98%
SP-Fitness → SP-Health → Overall QoL	0.12265	[0.07060, 0.17867]	<0.001	Large	42.34%
*With covariates (Age + Gender)*	0.00141	[−0.00131, 0.00357]	0.280	Small	1.79%

ACME, Average Causal Mediation Effect; CI, Confidence Interval; SP, Self-Perceived.

#### Mediation effects across quality-of-life domains

Component-specific mediation analyses were conducted to determine whether SP health mediated the relationship between the PA level and individual QoL domains. No statistically significant indirect effects were observed across the examined QoL domains (Supplementary Table S1). All bootstrap confidence intervals included zero.

#### Summary of mediation findings

Overall, the results indicate that the PA level was not indirectly associated with QoL through SP health. Instead, perceptual variables played a central role. SP fitness showed a significant indirect association with QoL, primarily through its strong sequential relationship with SP health. These mediation patterns are illustrated in Figure 1.

### Hypothesis 2: moderating role of self-esteem in the indirect association between physical activity and quality of life

Hypothesis 2 proposed that self-esteem moderates the indirect association between the PA level and QoL through SP fitness. Consistent with the analytical approach for Hypothesis 1, moderated mediation analyses were conducted to examine whether the strength of the indirect effects differed across levels of self-esteem. Participants were classified into low, moderate, and high self-esteem groups. Analyses were performed on the full sample (*N* = 230) using the mediation package in R with 1,000 bias-corrected bootstrap resamples. Age and gender were included as covariates. Indirect effects were examined for overall QoL and for individual QoL domains.

#### Moderated mediation analysis: overall QoL

When SP health was the mediator, the indirect effect of the PA level on overall QoL was not statistically significant in any self-esteem group. ACMEs were small and non-significant across low, moderate, and high self-esteem levels, indicating no evidence that self-esteem moderated the PA → SP health → QoL pathway.

By contrast, when SP fitness was the mediator, a different pattern emerged. The indirect effect of the PA level on the overall QoL increased across self-esteem levels, showing a marginally significant effect in the low self-esteem group, a statistically significant effect in the moderate group, and a highly significant effect in the high self-esteem group (Table 2). The conditional indirect effects showed a progressively stronger pattern across increasing levels of self-esteem for the PA level → SP fitness → QoL pathway, whereas the corresponding indirect effects through SP health were not statistically significant.

**Table 2:** Conditional indirect effects of physical activity level on overall quality of life through self-perceived health and self-perceived fitness across self-esteem levels.

Self-esteem group	Mediator	ACME	95% CI	*p*-value	Significant?	Effect size
Low	Self-Perceived Health	0.00286	[−0.00468, 0.00994]	0.450	No	Small
Low	Self-Perceived Fitness	0.00606	[−0.00001, 0.01410]	0.054	Marginal	Medium
Moderate	Self-Perceived Health	0.00182	[−0.00180, 0.00521]	0.252	No	Small
Moderate	Self-Perceived Fitness	0.00238	[0.00004, 0.00562]	0.042	Yes	Small
High	Self-Perceived Health	−0.00414	[−0.01608, 0.00142]	0.202	No	Small
High	Self-Perceived Fitness	0.00677	[0.00248, 0.01334]	< 0.001	Yes	Medium

#### Moderated mediation across QoL domains

**Figure 2:**
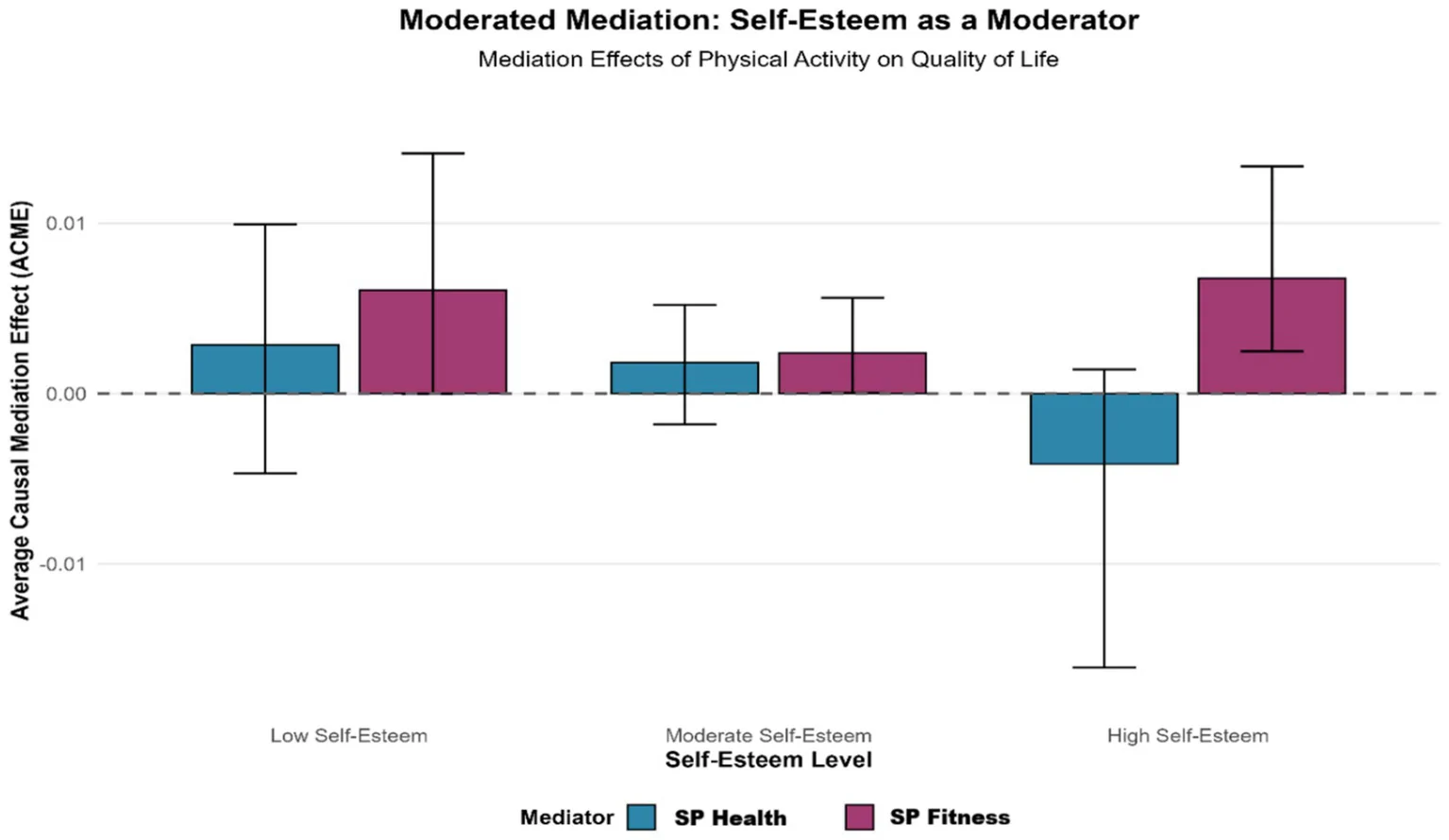
Moderated mediation by self-esteem across QoL domains.

Analyses across individual QoL domains yielded a consistent pattern. The indirect effects of the PA level through SP health were non-significant across the examined domains and self-esteem levels, whereas mediation through SP fitness generally increased across self-esteem levels (Figure 2). These domain-specific findings are presented as secondary analyses.

#### Summary of Hypothesis 2 findings

Taken together, these results partially support Hypothesis 2. As observed in Hypothesis 1, perceptual variables are central in linking the PA level to QoL. Extending these findings, self-esteem moderates the mediation pathway through SP fitness, such that the indirect effect of PA on QoL becomes stronger at higher levels of self-esteem. By contrast, SP health is neither a mediator of this relationship nor influenced by self-esteem. These findings indicate that self-esteem may strengthen the PA → fitness perception → QoL pathway, while the health perception pathway remains unaffected.

### Hypothesis 3: integrated model prediction of quality of life

Hypothesis 3 tested whether an integrated model combining the PA level, SP health, SP fitness, and self-esteem would significantly explain variance in QoL. A hierarchical multiple regression analysis was conducted, with predictors entered in sequential blocks to examine their incremental contribution.

As shown in Table 3, in the baseline model, the PA level alone explained virtually no variance in QoL. The addition of the self-perception variables, SP health and SP fitness, resulted in a substantial and statistically significant improvement in model fit (*p* < 0.001), accounting for 27.31% of the variance. Adding self-esteem led to a minimal and non-significant increase (*p* = 0.315). Finally, the interaction model, which included significant interaction terms among predictors, accounted for the largest proportion of explained variance (R^2^ = 0.3862), with the improvement driven primarily by interactions involving self-perception variables.

**Table 3:** Hierarchical regression analysis predicting quality of life from the physical activity level, self-perception variables, and self-esteem.

Model	Predictors included	*R* ^2^	Δ*R*^2^	*F*-change	*p*-change
1. Baseline	PA level only	0.0000	–	–	–
2. Add self-perceptions	PA level + self-perceived health + self-perceived fitness	0.2731	0.2731	42.46	<0.001
3. Add self-esteem	Full, integrated model (all main effects)	0.2764	0.0033	1.02	0.315
4. With interactions	Best-fitting interaction model	0.3862	0.1097	13.23	<0.001

*N* = 230; Δ*R*^2^ = change in explained variance from the previous model.

**Figure 3:**
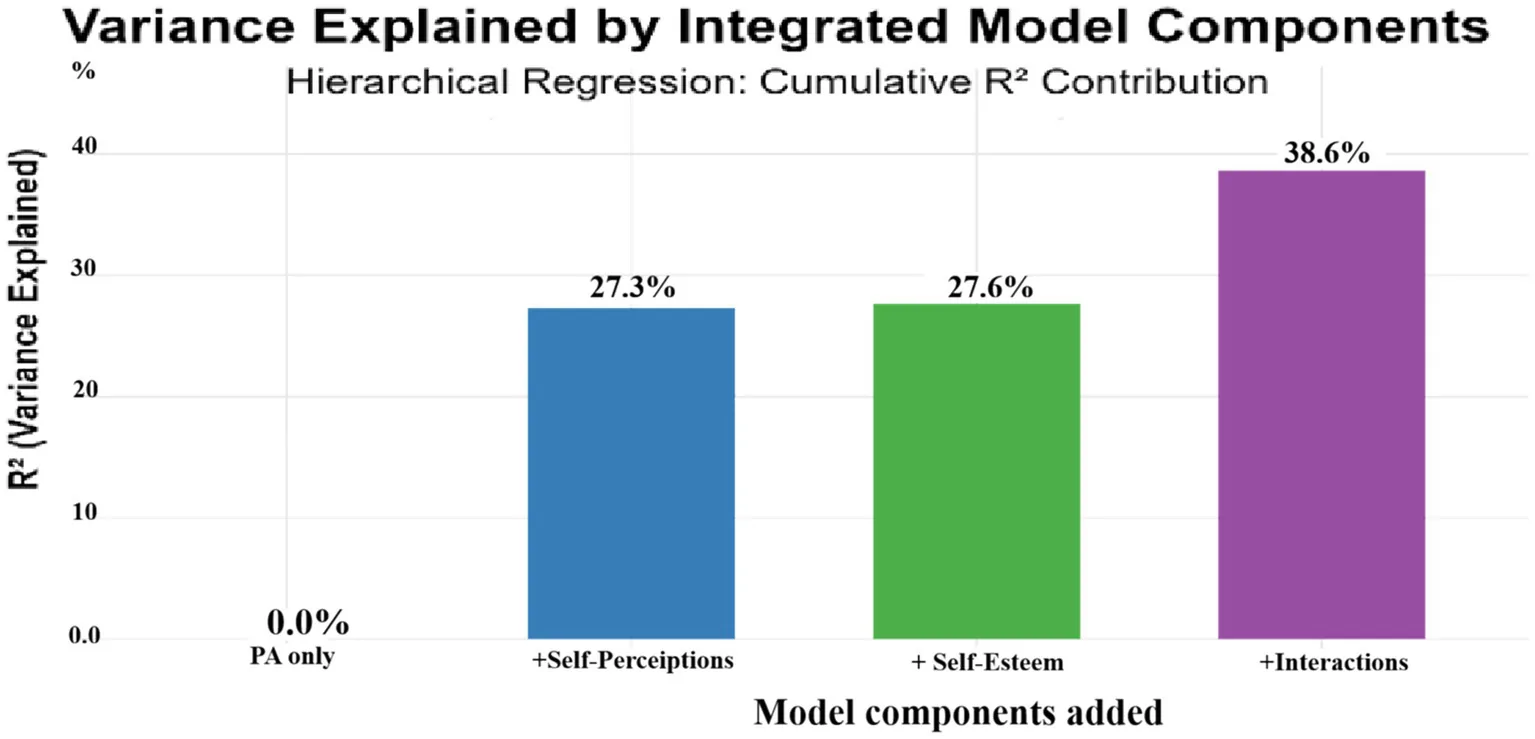
Cumulative variance in QoL explained by sequential predictor blocks.

The regression coefficients for the final main-effects model (Model 3) are shown in Table 4. SP health emerged as the strongest predictor of QoL (*β* = 0.431, p < 0.001), followed by SP fitness (*β* = 0.203, *p* = 0.001). By contrast, neither the PA level (*β* = −0.073, *p* = 0.215) nor self-esteem (*β* = −0.067, *p* = 0.314) showed a statistically significant direct association with QoL. Overall, the final main-effects model explained 27.6% of the variance in QoL, with an adjusted R^2^ of 26.4% (Figure 3).

**Table 4:** Regression coefficients for the final integrated model predicting quality of life.

Predictor	*B* (Unstandardized)	*SE*	*β* (Standardized)	*t*-value	*p*-value	95% CI
Intercept	2.959	0.163	–	18.130	<0.001	[2.638, 3.280]
PA level	−0.0035	0.0028	−0.073	−1.243	0.215	[−0.0090, 0.0020]
Self-perceived health	0.447	0.062	0.431	7.178	<0.001	[0.325, 0.569]
Self-perceived fitness	0.185	0.056	0.203	3.273	0.001	[0.074, 0.296]
Self-esteem	−0.0059	0.0058	−0.067	−1.008	0.314	[−0.0174, 0.0056]

Model *R*^2^ = 0.2764, Adjusted *R*^2^ = 0.2636, *F* (4, 225) = 21.49, *p* < 0.001. CI, confidence interval.

To assess model robustness, cross-validation was conducted using a 70/30 training–test split. The integrated model demonstrated stable predictive performance on the test dataset (*R*^2^ = 26.8%, root mean squared error = 0.350, mean absolute error = 0.293, mean absolute percentage error = 9.1%), indicating good generalizability. These results suggest that the model provides reliable predictions, with average prediction errors below 10% of the observed QoL scores.

## Discussion

The present study addressed the psychological processes mediating the relationship between PA and QoL in IWPDs. The results corroborate a theory-driven statistical model that included behavioral PA level with perceptual dimensions (self-perceived fitness, self-perceived health, and self-esteem) and where subjective assessments were engaged in the association between PA and QoL. The model is theoretical and cross-sectional; hence, no inferences on temporal order or causality can be made. A detailed description of the perception-centered model is provided in the Theoretical Integration section.

### The centrality of perceptual pathways (Hypothesis 1)

The results indicate that sequential cognitive appraisal was statistically associated with the PA–QoL, rather than the relationship being explained solely by direct physiological mechanisms. The notable sequential indirect association/pathway, where SP fitness indirectly linked to QoL through SP health, constituted 42.3% of the total effect, indicating a structured perceptual hierarchy. This suggests that individuals may assess their activities based on capability and mastery (fitness), and this specific perception is subsequently associated with broader health evaluations, which in turn are associated with QoL.

The findings extend the application of social cognitive theory (Bandura, 1997) by delineating a cascade from domain-specific to domain-general within self-referent thought. They provide a possible explanation for the “disability paradox” (Albrecht and Devlieger, 1999), in which subjective well-being frequently remains elevated despite considerable functional impairments. The model suggests that this pattern may be related to well-being being rooted in adaptive self-perceptions of fitness and health, rather than in objective biomedical or behavioral metrics. This is consistent with the biopsychosocial model outlined in the International Classification of Functioning, Disability, and Health (World Health Organization, 2001), in which cognitive appraisal may represent a link between body function/activity and participation/well-being.

The prominence of SP health as the most significant direct predictor of QoL supports a substantial body of epidemiological research indicating that subjective health assessments frequently exceed objective measures in forecasting important outcomes (Benyamini and Idler, 1999; Idler and Benyamini, 1997). Our findings indicate that, within the realm of PA, this significant subjective evaluation is associated with earlier, more specific perceptions of fitness. The common-sense model (Leventhal et al., 2003) has been widely used in the study of illness representations. Our findings may illustrate its applicability in understanding health behavior representations, wherein the cognitive-emotional processing of an individual’s activities may be related to a “fitness representation” that is associated with coping strategies and outcomes.

The absence of a clear connection from PA level to QoL through SP health does not align with basic biomedical ideas that assume a direct relationship. This finding suggests that objective behavioral engagement alone was not sufficient to account for variation in well-being; rather, the observed associations involved subjective registration and positive appraisal through perceptual filters. The findings are consistent with response shift theory (Sprangers and Schwartz, 1999), indicating that the underlying process may involve reconceptualization. IWPDs may adaptively redefine concepts such as “fitness” and “health” based on personal capability and mastery, thus potentially distinguishing QoL from conventional performance standards.

### The moderating role of self-esteem (Hypothesis 2)

Data relative to Hypothesis 2 refined the model by identifying self-esteem as a significant yet selective moderator. Figure 1 illustrates that self-esteem moderates the indirect relationship between PA level and QoL via SP fitness, with effects intensifying from low to moderate to high self-esteem groups. This graded pattern aligns with self-consistency theory (Swann, 2011), suggesting that individuals with higher self-esteem tend to interpret their behavior positively and in a self-congruent manner, thereby strengthening the observed association between activity and positive fitness appraisal.

Moreover, self-esteem did not serve as a moderator in the relationship involving SP health. This dissociation highlights the psychological difference between the two mediators. Fitness perceptions are intricately linked to assessments of mastery and competence, which are fundamental aspects of self-esteem (Bandura, 1997). By contrast, global health perceptions may encompass a broader range of factors, including those considered less dependent on personal agencies (e.g., genetic predisposition and chronic condition status), which may make them less strongly associated with global self-worth. This finding builds upon the research of McAuley et al. (2006) regarding self-efficacy by identifying global self-esteem as a contextual factor associated with differences in the strength of the observed relationship between behavioral PA level and positive self-perception.

### Predictive dominance of self-perceptions (Hypothesis 3)

Results of the hierarchical regression analysis (Hypothesis 3) provided essential convergent evidence consistent with the perceptual pathways model by clarifying the statistical roles and predictive hierarchy of its key components. The results demonstrated that SP health and SP fitness were significant predictors of QoL, whereas the PA level had a small direct statistical association with QoL. Furthermore, the analysis identified a clear hierarchical relationship between the perceptual mediators: SP health emerged as a substantially stronger direct predictor than SP fitness. This hierarchy is consistent with the possibility that domain-general health appraisal serves as a more proximal determinant of well-being than domain-specific fitness appraisal. Finally, the analysis clarified the distinct role of self-esteem: in contrast to its moderating role identified in Hypothesis 2, its non-significant direct effect in the full model is consistent with its role as an upstream contextual amplifier of the initial perceptual link rather than as a direct contributor to QoL variance.

This predictive dominance of self-perceptions over behavioral PA levels provides convergent evidence consistent with the importance of appraisal processes. The finding that PA level explained a small variance in QoL, whereas SP health and fitness accounted for a substantial 27.3%, aligns with the well-established “discrepancy hypothesis” in well-being research (Diener et al., 2006). This principle holds that subjective evaluations of one’s state are frequently stronger predictors of psychological outcomes than objective indicators. Our findings extend this principle to the disability context, suggesting that, even when objective functional capacity is central to daily life, the *perceived meaning* of one’s activity and health may be more closely associated with QoL than objective behavioral indicators. This pattern echoes findings in chronic illness research, where SP health consistently outperforms clinical metrics in predicting well-being and healthcare utilization (Fayers and Sprangers, 2002).

The hierarchical relationship between SP fitness and health is consistent with hierarchical models of self-concept. Marsh’s (1990) multidimensional self-concept theory asserts that evaluations in specific domains (e.g., physical fitness) combine to create broader domain self-concepts (e.g., physical self-worth), which in turn affect overall self-esteem. The findings are consistent with a similar structure in health perceptions, in which the specific assessment of fitness competence is associated with the broader construct of perceived health, which subsequently shows the strongest statistical association with overall QoL. This pattern of indirect associations provides a possible explanation for how domain-specific activity appraisals may relate to overall well-being.

The non-significant direct contribution of self-esteem in the full model contrasts with its significant moderating role in H2, providing important theoretical clarification. This pattern corresponds with the debate between “bottom-up” and “top-down” approaches in well-being research. Our findings suggest that, within this cross-sectional model, the relationship is primarily “bottom-up”: domain-specific self-perceptions (fitness, health) show stronger statistical associations with QoL, while global self-esteem may function as a schema filter associated with the initial interpretation of objective experiences (Swann, 2011). This may help clarify a discrepancy in the literature regarding the role of self-esteem as a predictor of well-being versus a moderator. It suggests that its influence may be dependent on the level of analysis, with direct effects decreasing when domain-specific mediators are considered.

The significant explanatory contrast highlights the practical implications of measurement. The performance of the model supports the need for a balanced assessment paradigm in rehabilitation science that emphasizes patient-reported outcomes in conjunction with performance-based measures (Dijkers, 2003). Our findings suggest that, in clinical contexts involving IWPDs, monitoring variations in SP fitness and health could better understand well-being changes compared with solely tracking changes in objective activity counts.

### Theoretical integration: toward a perception-centered model

The findings of this study are consistent with a comprehensive perception-centered model of well-being for IWPDs. The findings suggest that behavioral PA level shows a small, statistically non-significant direct association with QoL. Instead, its associations with QoL are observed through a series of distinct perceptual pathways, involving behavior-specific perceptions (SP fitness) and domain-general perceptions (SP health).

These pathways vary according to self-esteem, with stronger indirect associations observed at higher levels of self-esteem in the initial behavioral assessment but not in later health evaluations. Both SP health and SP fitness emerged as significant statistical correlates of QoL, with SP health showing the strongest association. This model synthesizes social cognitive theory (Bandura, 1997), the common-sense model (Leventhal et al., 2003), and self-consistency principles (Swann, 2011); it describes the pattern of the observed perceptual associations and highlights their stronger statistical relationship with QoL compared with behavioral PA level in the context of well-being. This perception-centered model is constrained to a psychological understanding of PA. It does not refute the significance of physiological improvements or environmental factors (as highlighted in the ICF framework); instead, it focuses on how individuals’ perceptions of fitness and health may be associated with QoL. While Wilson and Cleary's (1995) model proposed a general pathway from clinical variables to perceived health and QoL, our results complement and extend this model by highlighting distinct roles for behavior-specific fitness perceptions and domain-general health perceptions and by positioning self-esteem as a contextual moderator of the initial behavioral interpretation. Unlike the ICF, which provides a broader framework encompassing bodily functions, activities, participation, and contextual factors, our model focuses specifically on self-evaluation and perceptual processes that may complement these domains when explaining QoL in this demographic.

### Limitations and future directions

The current study has several limitations that should be acknowledged. First, the cross-sectional design and the exclusive use of self-reported measures limit the ability to verify the temporal ordering assumed in the mediation model. Although the proposed directionality was theory-driven, longitudinal or experimental studies are needed to establish temporal precedence and causality. Second, the sample was limited to individuals with physical disabilities, potentially restricting the generalizability of the findings. The experience of living with a physical disability may influence the perception of physical activity, fitness, health, and quality of life, which calls for replication in more diverse populations. Third, self-perceived health and self-perceived fitness were assessed using single-item measures, which may not capture these constructs as comprehensively as multi-item instruments. Future studies should consider using validated multidimensional scales. Fourth, PA was assessed using the validated self-report PASIPD-AR questionnaire. Although this instrument has demonstrated satisfactory psychometric properties, self-reported measures are inherently subject to recall and social desirability biases and may not fully capture the spectrum of PA, particularly light-intensity activities and activities of daily living. Future studies should consider complementing self-reported assessments with device-based measures, such as accelerometers, where feasible. Finally, self-esteem was categorized into three levels to facilitate the moderated mediation analyses, which may have reduced statistical power and obscured variability within the original continuous measure. Moreover, the static nature of our analysis does not capture potentially dynamic and reciprocal associations among self-esteem, self-perceptions, and quality of life. Employing intensive longitudinal methods would better understand these processes over time.

Examine the hypothesized serial indirect pathway (PA level → SP fitness → SP health → QoL) through longitudinal designs, as this unified indirect effect was not directly estimated in the present study.

Examine further moderators and mediators, including disability type, social support, self-efficacy, and autonomy as outlined in self-determination theory (Ryan and Deci, 2000).

Design and assess interventions aimed at improving positive self-perceptions related to activity and health, in conjunction with promoting behavioral changes.

### Practical implications

This study suggests that interventions for IWPDs could encompass both the enhancement of PA and the promotion of positive cognitive appraisal. Programs could assist participants in acknowledging and internalizing their activities as indicators of personal fitness, employing strategies such as positive feedback, cognitive restructuring, and self-monitoring. The findings emphasize the significance of connecting perceived fitness to overall health, which may allow individuals to develop a narrative in which activity plays a meaningful role in their well-being. Individuals with lower self-esteem may require initial support through strengths-based approaches, mastery-oriented goal setting, or self-compassion training to promote positive activity interpretations. In conclusion, our findings suggest that clinical and research evaluations should regularly incorporate validated assessments of SP fitness and health, as these subjective perceptions showed stronger statistical associations with QoL than PA in the present study.

## Conclusion

This study indicates a strong association between psychological factors and the relationship between PA and QoL among IWPDs. Collectively, the findings support a perception-centered model of well-being in this population. PA level alone showed a small, statistically non-significant association with QoL, whereas SP fitness and health showed stronger statistical associations with QoL and significant indirect associations in the tested models. Self-esteem was associated with stronger indirect associations through the fitness perception pathway, highlighting the psychological translation of behavior as an important correlate of well-being. Interventions could therefore consider targeting not only behavioral engagement but also subjective appraisals of capability and health as potential components for supporting QoL.

## Data Availability

The raw data supporting the conclusions of this article will be made available by the authors, without undue reservation.
