# Supplementary material for: The mediating role of self-perceived health and fitness in the relationship between physical activity and quality of life among individuals with physical disabilities: the moderating effect of self-esteem
Source: Front Psychol. 2026 Aug 27;17:1784783. doi: 10.3389/fpsyg.2026.1784783 (PMC13558094; doi:10.3389/fpsyg.2026.1784783)
Supplement: Supplementary file 1 [file Table_1.DOCX]

**Table S1.** *Mediation Effects of the Physical Activity Level on Specific Quality of Life Domains via Self-Perceived Health*

| **QoL Domain** | **ACME** | **95% CI** | ***p*-value** | **Significant?** |
| --- | --- | --- | --- | --- |
| Physical Health | 0.00222 | [−0.00566, 0.00897] | .504 | No |
| Psychological Health | 0.00128 | [−0.00315, 0.00524] | .504 | No |
| Social Relationships | 0.00060 | [−0.00156, 0.00297] | .514 | No |
| Environment | 0.00081 | [−0.00238, 0.00297] | .504 | No |
| Discrimination | 0.00165 | [−0.00431, 0.00657] | .504 | No |
| Autonomy | −0.00030 | [−0.00267, 0.00116] | .714 | No |
| Inclusion/Participation | 0.00003 | [−0.00058, 0.00068] | .940 | No |
